# Re-evaluation of population-level protection conferred by a rotavirus vaccine using the ‘fried-egg’ approach in a rural setting in Bangladesh

**DOI:** 10.1016/j.vaccine.2021.08.048

**Published:** 2021-09-24

**Authors:** Asma Binte Aziz, K. Zaman, Deok Ryun Kim, Ju Yeon Park, Justin Im, Mohammad Ali, Faisal Ahmmed, Md Taufiqul Islam, Farhana Khanam, Fahima Chowdhury, Tasnuva Ahmed, Masuma Hoque, Xinxue Liu, Gi Deok Pak, Birkneh Tilahun Tadesse, Hyon Jin Jeon, Sophie Kang, Ashraful Islam Khan, Jerome H. Kim, Florian Marks, Firdausi Qadri, John David Clemens

**Affiliations:** a International Centre for Diarrhoeal Disease Research, Bangladesh (icddr,b), Dhaka, Bangladesh; b International Vaccine Institute, 08826 Seoul, Republic of Korea; c Institute of Clinical Medicine, University of Oslo, Norway; d Cambridge Institute of Therapeutic Immunology and Infectious Disease, University of Cambridge School of Clinical Medicine, Cambridge, United Kingdom; e Johns Hopkins University, Baltimore, MD, USA; f University of Oxford, Oxford, United Kingdom; g University of Antananarivo, Antananarivo, Madagascar; h UCLA Fielding School of Public Health, Los Angeles, CA 90095-1772, USA

**Keywords:** Rotavirus, Rotavirus vaccine, Fried-egg, Herd protection, CRT

## Abstract

**Background:**

Vaccine herd protection assessed in a cluster-randomized trial (CRT) may be masked by disease transmission into the cluster from outside. However, herd effects can be unmasked using a ‘fried-egg’ approach whereby the analysis, restricted to the innermost households of clusters, ‘yolk’, creates an insulating ‘egg-white’ periphery. This approach has been demonstrated to unmask vaccine herd protection in reanalyses of cholera and typhoid vaccine CRTs. We applied this approach to an earlier CRT in Bangladesh of rotavirus vaccine (RV) whose overall analysis had failed to detect herd protection. Herein we present the results of this analysis.

**Methods:**

In the study area, infants in 142 villages were randomized to receive two doses of RV with routine EPI vaccines (RV villages) or only EPI vaccines (non-RV villages). We analyzed RV protection against acute rotavirus diarrhoea for the entire cluster (P100) and P75, P50, P25 clusters, representing 75%, 50% and 25% of the innermost households for each cluster, respectively.

**Results:**

During 2 years of follow-up, there was evidence of 27% overall (95 %CI: 7, 43) and 42% total protection (95 %CI: 23, 56) in the P100 cluster, but it did not increase when moved in smaller yolks. There was no evidence of indirect vaccine protection in the yolks at any cluster size.

**Conclusion:**

Our reanalysis of the CRT using the fried- egg approach did not detect RV herd protection. Whether these findings reflect a true inability of the RV to confer herd protection in this setting, or are due to limitations of the approach, requires further study.

## Highlights

• The “fried-egg” analytic approach was applied to a cluster randomized trial (CRT).
• Overall analysis failed to reveal rotavirus vaccine (RV) herd protection.
• Same approach unmasked herd protection of other enteric vaccines failed for RV.

## 1 Introduction

Rotavirus infection is a major cause of diarrhoeal morbidity and mortality in children under 5 years of age and leads to an estimated 215,000 (197,000–233,000) deaths globally [1]. Currently, four rotavirus vaccines (RVs) (ROTARIX, GSK; RotaTeq, Merck; ROTAVAC, Bharat Biotech; and ROTASIIL, Serum Institute of India) are included in the national immunization programs of 110 countries worldwide [2]. The protection afforded by these vaccines, however, has been reported to be systematically lower in low-income countries when compared to high-income settings [3]. Thus, in low-income countries, where the risk of rotavirus infection is higher, herd protective effects may be of particular importance in containing disease spread [4]. However, the evidence for RV herd protection is strongest in more affluent countries [5], [6], [7]. A cluster-randomized trial (CRT) using a RV, Rotarix (GSK) was conducted in Matlab, Bangladesh between 2008 and 2011 [8]. There was no evidence of vaccine herd protection in this trial using a conventional analytical approach [8]. We reasoned that this could have occurred due to the possible transmission of rotavirus from outside the clusters, as there were no impermeable boundaries between the clusters. Herein we present a reanalysis of the trial, using the “fried-egg” analytic approach, intended to compensate for this bias [9].

## 2 Methods

### 2.1 The study area

The trial was conducted in Matlab, a rural field study area of the icddr,b (formerly known as the International Centre for Diarrhoeal Disease Research, Bangladesh) close to Dhaka, Bangladesh. The population under analysis has been observed under a Health and Demographic Surveillance System (HDSS) since 1966 and is well-described through routinely updated population census activities [10]. The study area comprises 142 villages geographically divided into two administrative areas, the icddr,b service area (ISA) made up of 67 villages, and the government service area (GSA) made up of 75 villages. In ISA, icddr,b provides enhanced services, including maternal, child health, and family planning services and routine immunization; in GSA, the Bangladesh Ministry of Health and Family Welfare provides standard public health and routine immunization services. Geographic coordinates are recorded for each household. The residents of Matlab predominantly belong to low- and middle-income communities and small-scale farming and fishing are the main occupations. The routine Expanded Programme on Immunization (EPI) vaccines were Bacillus Calmette-Guerin (BCG), Oral poliovirus Vaccine (OPV), diphtheria-tetanus-pertussis (DTP) and measles, administered at 6 weeks, 10 weeks, 14 weeks and 9 months of age during the study period [8].

### 2.2 Vaccination

The cluster-randomized trial evaluated Rotarix (GSK Biologicals, Rixensart, Belgium), an attenuated monovalent RV [11], which was delivered by the EPI staff in two oral doses separated by 4 weeks. Infants 6–20 weeks of age at the time of vaccination and residing in randomly assigned villages were offered the first dose of RV, given simultaneously with DTP1 or DTP2 vaccine delivered by the EPI in Bangladesh at 6 and 10 weeks of age. The second dose of RV was given 4 weeks after the first dose but before 20 weeks of age. Parents or legal guardians of participating infants provided written informed consent. Rotavirus vaccination, which was started in GSA from November 1, 2008 and in ISA from April 1, 2009, was carried out continuously during the study period. Vaccination status was recorded in the EPI cards as well as in the study forms. Infants in the non-RV villages received only the routine EPI vaccines [8].

### 2.3 Study population

A total of 142 villages within the Matlab HDSS were randomly assigned in a 1:1 ratio to either the RV study villages (total population 116,649) or non-RV study villages (total population 105,569). Infants who were 6–20 weeks of age on or after the study initiation date (November 1, 2008 in the GSA and April 1, 2009, in the ISA) and were included in the HDSS database were eligible for participation. Demographic and socioeconomic data were collected from this database. Individual vaccination records were also retrieved and linked to the database. The geographic linear distances (km) from the child’s residence to the Matlab icddr,b hospital, was calculated using geographic information system (GIS) data collected as part of the HDSS.

### 2.4 Diarrhoea surveillance

Diarrhoea surveillance was conducted at the main icddr,b hospital in Matlab as well as in two community-operated treatment centers run by icddr,b in Matlab, the only sources of care for diarrhoea for the trial population. Children less than 2 years of age presenting to any of these health facilities with diarrhoea were assessed and treated accordingly. Diarrohea was defined as having three or more loose stools within 24 h of presentation; severe dehydration was defined as per WHO criteria [12]. Stool samples were collected from all patients coming from the trial area and were tested for rotavirus at the icddr,b laboratory. An acute rotavirus diarrhoea (ARD) was defined as a diarrhoea patient with symptoms less than 7 days in duration and whose stool specimen yielded rotavirus antigen (group A rotavirus specific VP6 proteins) using a qualitative enzyme immunoassay (Prospect Rotavirus Microplate Assay, Oxoid, Basingstoke, UK). Clinical characteristics of illnesss, treatment history and health outcome were recorded in the surveillance system [8]. Acute rotavirus gastroenteritis can also be characterized only by emesis (≥ 1 episode of forceful vomiting within 24 h), but this is rare in Bangladesh. Our analysis is a reanalysis of an earlier trial of the GSK's RV, which, like other trials of this vaccine, targeted diarrheal disease as the primary outcome. The trial was completed on March 31, 2011.

### 2.5 Analytic approach

**Fig. 1:**
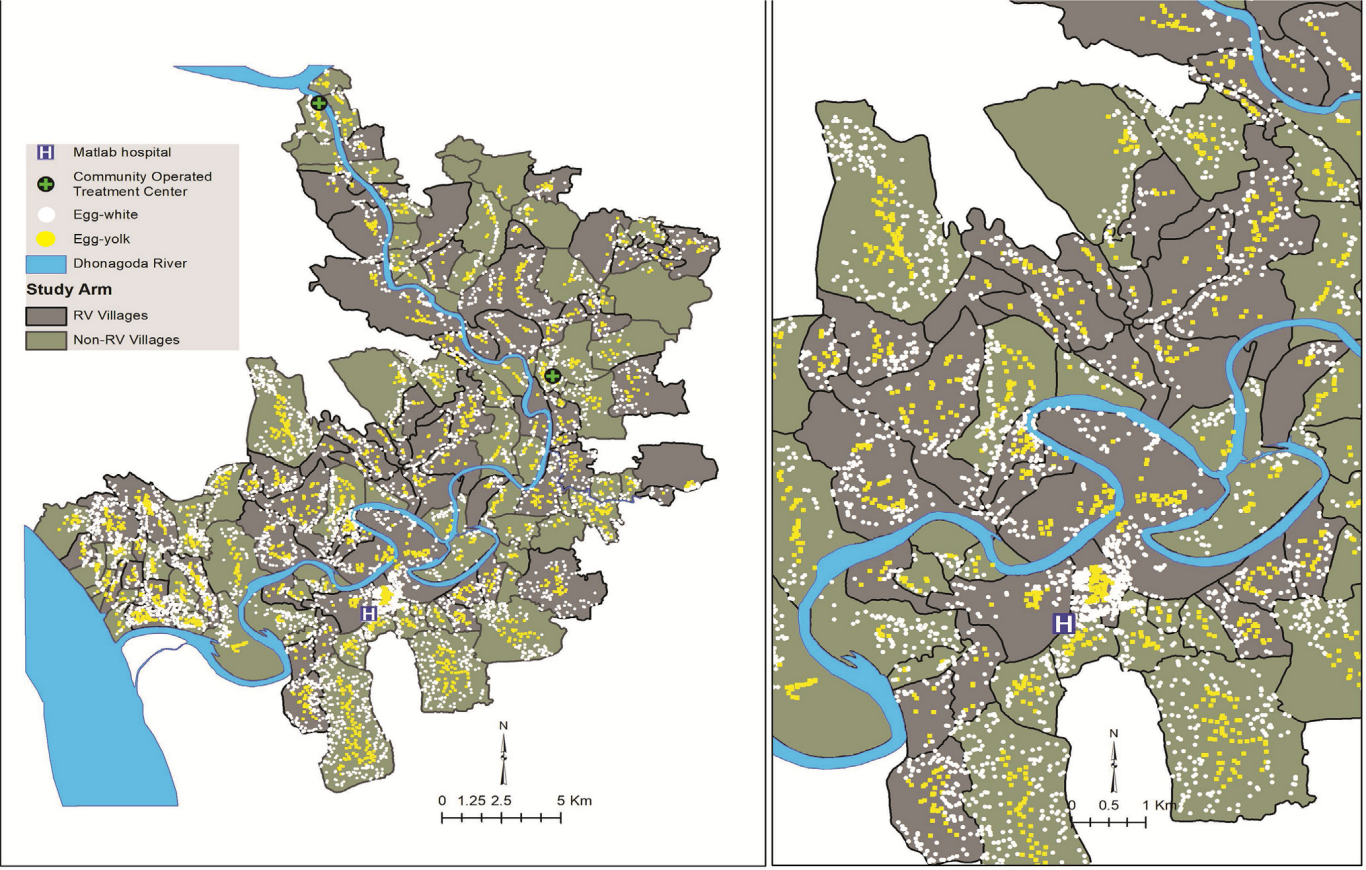
Distribution of study area households for P25 clusters (left:entire study area; right:magnified view of P25).

We used a ‘fried-egg’ approach [9] to reanalyze the Matlab CRT dataset. In this approach, progressively smaller central ‘yolks’ within the original allocation clusters demarcated the study population are analysed for evidence of vaccine herd protection. The logic is that as the yolks become smaller and are hence surrounded by wider ‘whites’ of surrounding populations, these surrounding populations would progressively insulate the yolk populations from the inward transmission of rotavirus, thereby revealing true vaccine herd protective effects. We computed the distance (linear) from the child’s residence to the nearest village boundary. To define the different size of yolks, we first sorted the distances from the village boundaries in descending order (furthest from to closest to the nearest village boundary) within the cluster. We then assembled successive proportions of the study population, beginning with the household furthest from the village boundary and proceeding to include households progressively closer to the nearest village boundary until the desired fraction of households in the cluster was achieved. We specified four fractions of households for analysis: 25% households (innermost yolk), 50% households, 75% households, and 100% households (outermost including the entire cluster) referred to, respectively as P25 clusters, P50 clusters, P75 clusters, and P100 clusters [13]. We hypothesized that if herd protection was attenuated by the transmission of rotavirus into the clusters from the outside, this protection would be most evident in the innermost households. Fig. 1 shows households that were included in analyses of overall rotavirus protection for the P25 clusters.

### 2.6 Statistical analysis

Three different measurements of population-level vaccine protection were estimated: overall protection (protection of all children in the RV clusters relative to control residents), total protection (protection of RV recipients relative to control residents who had received OPV), and indirect protection (protection of non-RV recipients in RV clusters relative to control participants). All measures of vaccine protection against ARD were expressed as the proportionate reduction of rotavirus disease incidence in RV versus non-RV villages.

For overall protection, children who were 6–20 weeks old at study initiation, migrated in at 6–20 weeks of age during the study period, or became 6 weeks old during the study period were considered for analysis regardless of whether they received a vaccine (RV/ OPV) or not. The start date for follow-up was defined as the earliest of these three dates for each analyzed child.

For total protection, among all children who were eligible for overall protection, we analyzed only those who received at least one dose of RV in RV villages and received at least one dose of OPV in non-RV villages and did not receive the first dose prior to the study initiation. Start date of follow-up was the date of vaccination with RV or OPV.

For indirect protection, children who were aged 20 weeks to 24 months at study initiation or who migrated in between 20 weeks and 24 months of age and who but who did not receive RV as per record in the EPI card were included in the analysis. The start date of observation was defined as the date of initiation of the study, date of migration into the study villages, or date of aging into the targeted age range, whichever came first.

The end of follow-up for all measures of protection was considered as the date of turning 23.9 months of age, migration out of the study area, death, the study end date, or development of ARD, whichever came first. Person-years were calculated as the sum of individual periods of observation for the population under analysis.

To estimate vaccine protection, we analyzed the time to first episode of ARD, fitting Cox proportional hazards regression models with village vaccine assignment, together with potentially confounding covariates, including sex, mother’s highest education, house wall construction with bricks, TV ownership, reported usage of a modern toilet at home, use of a tubewell as the source of drinking water (a type of water well in which a long, 100–200 mm-wide, stainless steel tube or pipe is bored into an underground aquifer and water is pumped out through a handle present on it), and distance of household to the icddr,b Matlab hospital (km), as independent variables. Covariates were identified by backward selection with a cut-off p-value of <0.05 when compared between RV and non-RV villages for differing yolk sizes. Hazard ratios (HR) were estimated by exponentiation of the coefficient for the village vaccine assignment variable in models and vaccine protection was estimated as [(1 − HR) × 100%]. Standard errors for the coefficients were used to estimate two-tailed p-values and 95% confidence intervals for the HRs. All statistical analyses were performed using SAS version 9.4.

### 2.7 Role of the funding source

The initial trial was funded by Gavi, the Vaccine Alliance (GAV.1141–02) through PATH's Rotavirus Vaccine Program (RVP). GSK donated the vaccine for the study. None of the funding agencies were involved in the study design, data collection, analysis, interpretation or writing of the manuscript. The corresponding author had full access to all the data sets. This publication was made possible through a grant from the Bill & Melinda Gates Foundation (INV-025388).

### 2.8 Ethics

The original protocol was approved by the Instutional Review Board of icddr,b and the Western Institutional Review Board. The protocol was registered at ClinicalTrials.gov (NCT00737503).

## 3 Results

**Fig. 2:**
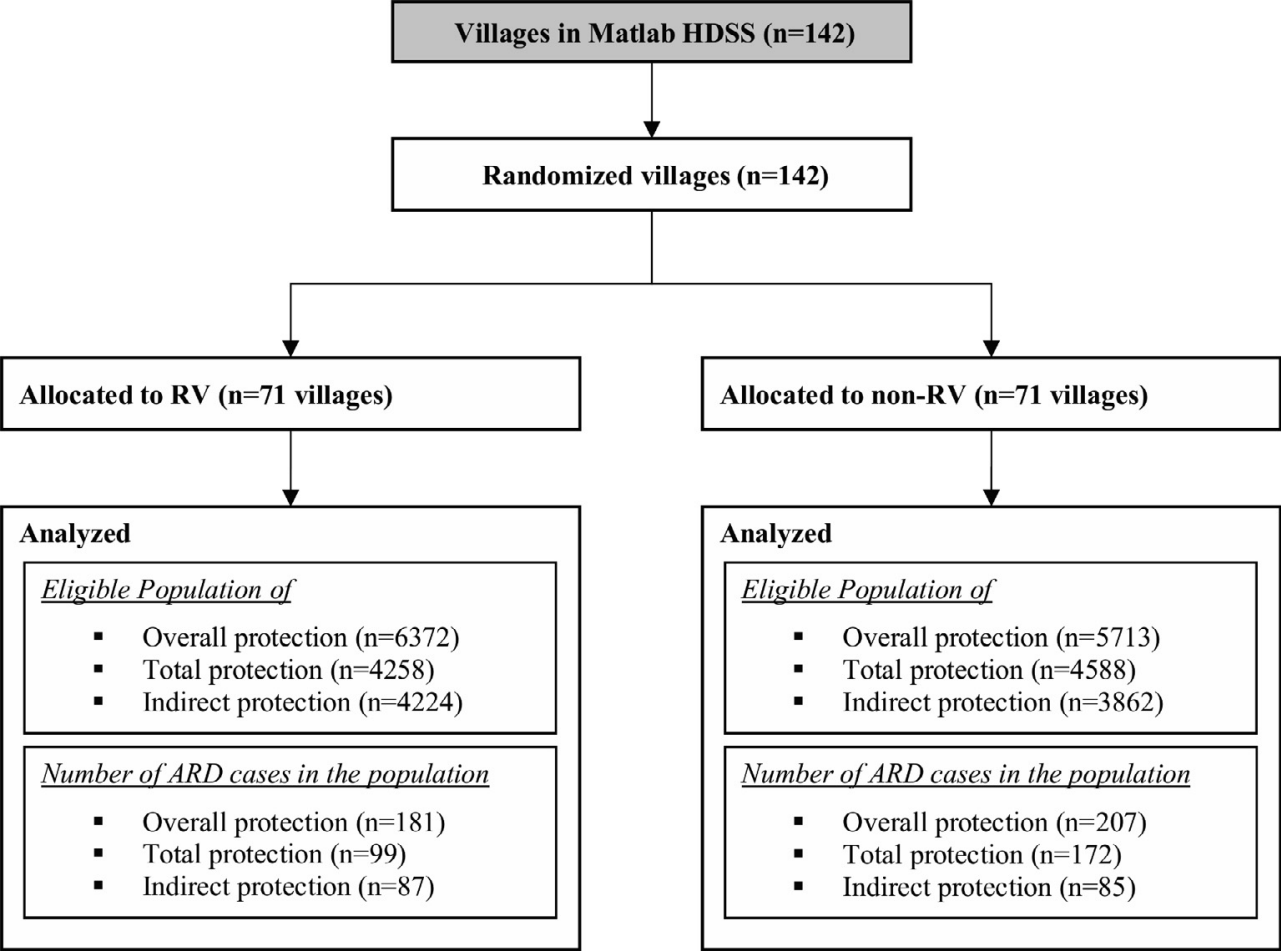
CONSORT diagram for analysis of overall, total and indirect vacine protection.

The RV and non-RV villages were well balanced with respect to baseline characteristics for analyses of overall (Table 1), total (Table 2**)** and indirect protection (Table 3) for P100 clusters as well as for the populations in the P75, P50 and P25 clusters (See Supplementary Tables). In RV villages, 4258 (67%) of 6372 age-eligible children to receive RV were vaccinated with at least one dose of RV. In non-RV villages, 4588 (80%) of 5713 children received at least one dose of OPV vaccine as a part of routine immunization program in Bangladesh and and were included in the analysis of total vaccine protection **(**Fig. 2**).** In the non-RV villages, which were served by the Government Health system, Government staff administered OPV even in older age schedules other than 6, 10, and 14 weeks. In contrast, in RV villages, served by staff of the icddr,b, OPV was administered more strictly, maintaining the routine schedule at 6, 10, and 14 weeks of age and RV at 6 and 10 weeks. That’s why there is lower uptake of OPV in RV villages. However, all villages were equally served by the diarrhea treatment centers run by the icddr,b.

**Table 1:** Baseline population characteristics for analysis of overall protection (P100 clusters).

Variables	*RV Villages (N = 6372)	**Non-RV Villages (N = 5713)
Mean (SD) age at the time of study initiation/migration-in (years)	0.1 (0.1)	0.1 (0.1)
Male participants (%)	3223 (51)	2906 (51)
Mother’s education (8-class and above) (%)	3062 (48)	2720 (48)
Live in a household with a Pacca roof (%)	211 (3)	161 (3)
Live in a household with a Television (%)	1362 (21)	1182 (21)
Live in a household with using Septic tank/Modern toilet (%)	553 (9)	439 (8)
Live in a household using tubewell for drinking water (%)	4551 (71)	4066 (71)
Median (IQR) distance (km) to the icddr,b Matlab hospital	6.0 (6.5)	5.5 (4.9)

Note: the level of significance was derived after adjusting for the design effect; no statistical significance (p < 0.05) was detected between RV and non-RV Villages.

* **RV Villages –** participants received Rotavirus vaccine.

** **Non-RV Villages –** participants received only the routine EPI vaccines.

**Table 2:** Baseline population characteristics for analysis of total protection (P100 clusters).

Variables	*RV Villages (N = 4258)	**Non-RV Villages (N = 4588)
Mean (SD) age at the time of vaccination date (years)*	0.2 (0.0)	0.2 (0.1)
Male participants (%)	2118 (50)	2336 (51)
Mother’s education (8-class and above) (%)	1995 (47)	2177 (47)
Live in a household with a Pacca roof (%)	115 (3)	124 (3)
Live in a household with a Television (%)	845 (20)	932 (20)
Live in a household with using Septic tank/Modern toilet (%)	327 (8)	356 (8)
Live in a household using tubewell for drinking water (%)	3049 (72)	3270 (71)
Median (IQR) distance (km) to the icddr,b Matlab hospital	6.4 (6.0)	5.4 (4.8)

Note: the level of significance was derived after adjusted for the design effect; *statistical significance (p < 0.05) between RV and non-RV Villages.

* **RV Villages –** participants received Rotavirus vaccine.

** **Non-RV Villages –** participants received only the routine EPI vaccines.

**Table 3:** Baseline population characteristics for analysis of indirect protection (P100 clusters).

Variables	*RV Villages (N = 4224)	**Non-RV Villages (N = 3862)
Mean (SD) age at the time of study initiation/migration-in (years)	1.2 (0.5)	1.2 (0.5)
Male participants (%)	2123 (50)	1974 (51)
Mother’s education (8-class and above) (%)	1865 (44)	1651 (43)
Live in a household with a Pacca roof (%)	174 (4)	111 (3)
Live in a household with a Television (%)	961 (23)	864 (22)
Live in a household with using Septic tank/Modern toilet (%)	405 (10)	299 (8)
Live in a household using tubewell for drinking water (%)	3232 (77)	2952 (76)
Median (IQR) distance to the icddr,b Matlab hospital (Kilometer)	6.0 (6.5)	5.6 (5.0)

Note: the level of significance was derived after adjusted for the design effect; no statistical significance (p < 0.05) was detected between RV and non-RV Villages.

* **RV Villages –** participants received Rotavirus vaccine.

** **Non-RV Villages –** participants received only the routine EPI vaccines.

In the P100 clusters, for the analysis of overall protection, there were 388 ARD cases during the observation period, 181 cases in the RV villages and 207 in the non-RV villages (Table 4). The adjusted estimate for overall protection was 27% (95% CI: 7, 43). For total protection analysis, there were 271 ARD cases, 99 in RV and 172 in non-RV villages, respectively. Total protection was estimated as 42% (95% CI: 23, 56). There was no evidence for indirect vaccine protection, 7% (95% CI: −28, 33).

**Table 4:** Overall, total and indirect RV protection up to 24 months of age against ARD in the differently defined clusters.

Measure of Protection	**RV Villages			***Non-RV Villages			Adjusted protection	
	N	ARD cases/Person-years of follow-up	Incidence rate per 100 person-years (95% CI)	N	ARD cases/Person-years of follow-up	Incidence rate per 100 person-years (95% CI)	Estimate (95% CI)	P-value*
**P100 Clusters**								
Overall	6372	181/6551	3 (2, 3)	5713	207/5751	4 (3, 4)	27 (7, 43)	0.01†
Total	4258	99/4170	2 (2, 3)	4588	172/4415	4 (3, 5)	42 (23, 56)	0.00ǂ
Indirect	4224	87/3111	3 (2, 3)	3862	85/2869	3 (2, 4)	7 (−28, 33)	0.64§
**P75 Clusters**								
Overall	5007	146/5176	3 (2, 3)	4463	155/4488	3 (3, 4)	22 (−1, 40)	0.06†
Total	3333	76/3286	2 (2, 3)	3594	131/3459	4 (3, 4)	41 (19, 57)	<0.01ǂ
Indirect	3331	67/2451	3 (2, 3)	3043	60/2249	3 (2, 3)	−1 (−49, 31)	0.95§
**P50 Clusters**								
Overall	3407	100/3526	3 (2, 3)	3044	101/3037	3 (3, 4)	18 (−12, 40)	0.21†
Total	2252	53/2206	2 (2, 3)	2463	90/2359	4 (3, 5)	38 (9, 58)	0.01ǂ
Indirect	2249	51/1654	3 (2, 4)	2067	43/1545	3 (2, 4)	−9 (−63, 27)	0.66§
**P25 Clusters**								
Overall	1778	51/1857	3 (2, 4)	1613	52/1634	3 (2, 4)	16 (−29, 45)	0.42†
Total	1162	25/1154	2 (1, 3)	1314	44/1281	3 (3, 5)	39 (−2, 63)	0.06ǂ
Indirect	1168	28/868	3 (2, 5)	1078	21/796	3 (2, 4)	−17 (−90, 28)	0.53§

* Two-tailed 95% CIs and p-values are given for the analyses.

** **RV Villages –** participants received Rotavirus vaccine.

*** **Non-RV Villages –** participants received only the routine EPI vaccines.

† Overall protection for P100 and P75 clusters was adjusted for the variables used to stratify the clusters for randomization, sex, household with a television, mother’s education, and distance to the icddr,b hospital. For the P50 clusters, analysis was adjusted for the variables used to stratify the clusters for randomization, age at the start date (years), sex, mother’s education, and distance to the icddr,b hospital. For the P25 clusters, analysis was adjusted for the variables used to stratify the clusters for randomization, sex, mother’s education, and distance to the icddr,b hospital.

ǂ Total protection for P100 and P75 clusters was adjusted for the variables used to stratify the clusters for randomization, sex, household with a television, mother’s education, and distance to the icddr,b hospital. For the P50 clusters, analysis was adjusted for the variables used to stratify the clusters for randomization sex, mother’s education, household with a Pacca roof, and distance to the icddr,b hospital. For the P25 clusters, analysis was adjusted for the variables used to stratify the clusters for randomization, sex, mother’s education, and distance to the icddr,b hospital.

§ Indirect protection for P100 and P75 clusters was adjusted for the variables used to stratify the clusters for randomization, age at the start date (years), sex, mother’s education, and distance to the icddr,b hospital. For the P50 clusters, analysis was adjusted for the variables used to stratify the clusters for randomization, age at the start date (years), mother’s education, and distance to the icddr,b hospital. For the P25 clusters, analysis was adjusted for the variables used to stratify the clusters for randomization, age at the start date (years), and distance to the icddr,b hospital.

In the P75 clusters, for the analysis of overall protection, there were 301 ARD cases, 146 cases in the RV villages and 155 cases in the non-RV villages. The adjusted estimate for overall protection decreased to 22% cluster (95% CI: −1%, 40%). For total protection there were 208 ARD cases, 76 in RV and 132 in non-RV villages. Total protection was 41% (95% CI: 19% and 57%). There was also no evidence for indirect vaccine protection , −1% (95% CI: −49, 31) **(**Table 4**)**.

In the P50 clusters, for the analysis of overall protection, there were 201 ARD cases, 100 cases in the RV villages and 101 cases in the non-RV villages. The adjusted estimate for overall protection decreased somewhat to 18% (95% CI: −12%, 40%). For total protection there were 143 ARD cases observed, 53 in RV and 90 in non-RV villages, respectively. Total protection further declined to 38% (95% CI: 9% and 58%). There was also no evidence for indirect vaccine protection (-9%; 95% CI: −63, 27) (Table 4).

In the innermost P25 clusters, for the analysis of overall protection, there were 103 ARD cases, 51 cases in the RV villages and 52 cases in the non-RV villages. The adjusted estimate for overall protection further decreased to 16% (95% CI: −29%, 45%). For total protection there were 69 ARD cases observed, 25 in RV and 44 in non-RV villages. Total protection remained similar to that observed in P50 clusters, 39% (95% CI: −2% and 63%). Like other clusters, there was no evidence for indirect vaccine protection, −17% (95% CI: −90, 28) (Table 4).

## 4 Discussion

Reanalysis of this dataset using a fried-egg approach showed that population-level protection by RV measured as overall or total protection did not increase progressively in the smaller yolk populations. We also found no evidence for indirect vaccine protection across all yolk sizes despite the notion that a larger insulating ‘egg white’ in the smaller yolk clusters would reduce transmission of rotavirus from outside the cluster.

Although CRTs offer the most unbiased design for evaluating vaccine herd protection [14], CRTs often allocate clusters that are not fully self-contained epidemiological units of transmission. This thereby compromises the ability of such trials to measure vaccine herd protection, which depends on the fulfillment of this assumption [15]. The fried-egg design is an analytic approach to addressing this problem. However, there are several important limitations that must be additionally considered when interpreting the findings of this study. Firstly, children residing in the egg-white areas of vaccinated clusters were vaccinated, whereas children in the whites of unvaccinated clusters were not. This potentially resulted in greater buffering of inward transmission to the contained yolks in vaccinated clusters, elevating estimates of vaccine population protection. However, this consideration would have augmented, not diminished, measured population level vaccine protection with smaller yolk sizes, and thereby makes our negative findings conservative. Because the villages for the study directly abutted on one another, the rotavirus-vaccinated villages were not, on average, surrounded entirely by unvaccinated populations, with the result that the intensity of inward transmission of rotavirus into these vaccinated villages may have been reduced to some degree. However, such reduction of transmission should have facilitated the ability of our fried-egg approach to reveal vaccine herd protection. This observation strengthens our negative findings for vaccine herd protection. Secondly, since only infants under 20 weeks were vaccinated whereas vaccine protection was based on all children under 24 months of age, the clusters assigned to RV had a considerable portion of the follow-up period with low vaccine coverage of the group aged under 24 months, especially since the study interval was only 2 years. Such lower coverage might have diminished vaccine herd protection. Thirdly, the geographic size of clusters was relatively small and therefore shielding provided by the outer populations may have been ineffective in reducing transmission into the inner yolk. Had clusters been geographically substantially larger, residents in the inner regions may have been better insulated from inward transmission and inner yolks might have exhibited RV herd protection.

There is mixed evidence of herd protection by RVs in low-income countries. Whereas studies from Zambia and South Africa showed no evidence of RV indirect protection [16], [17], studies conducted in Zimbabwe and Rwanda showed indirect protection by RV [18], [19]. A study in Malawi also demonstrated some evidence of indirect protection in infants <12 months, but not in older children following RV introduction into routine immunization programs for infants [20]. On the other hand, evidence for herd protection has been more consistently found in higher-income countries such as United States, Mexico, and countries in Latin America [21], [22].

The mechanisms for RV herd protection evidenced in wealthy countries but possibly absent or inconsistent in low-income settings may be related to several factors. Firstly, RV direct protection is higher in developed countries [3]. A vaccine with high direct protection may be more successful at interrupting transmission and therefore confer greater herd protection at a given vaccine coverage level compared to a vaccine with lower direct protection. Secondly, rotavirus is thought to have greater person-to-person transmissibility (R_0_) in low-income countries, manifested as a lower average age of infection in poor relative to wealthy countries. This may be due to certain shared features common in developing countries, including overcrowded living conditions, limited access to improved water sources, and lack of hygiene and sanitation facilities, inherent transmissibility of circulating viral strains notwithstanding [23]. A pathogen with a higher R_0_ may be able to better penetrate the egg-white and thus nullify the benefit of analyzing the yolk only. Estimates of R_0_ for rotavirus range from 23.3 to 191.0 [23], and in Bangladesh it is estimated to be 72.2 [23]. As well, the higher transmissibility of rotavirus in poor settings such as rural Bangladesh may have created a high level of anti-rotavirus natural immunity at a relatively young age in children, and thereby diluted measured vaccine protection, both direct and herd, in the age range evaluated in our analyses, In contrast, previous analyses of vaccines against cholera and typhoid, pathogens for which the R_0_ is much lower, 1.06–2.63 and 2.8 respectively [24], [25], have revealed vaccine herd protection when the fried-egg approach was applied [13], [26]. Moreover, vaccine-preventable pathogens with a high R_0_ are known to require higher levels of vaccine coverage to interrupt transmission [27]. For low-income settings such as Bangladesh, there may thus be inherent limitations in the ability of RV to confer vaccine herd protection. This higher R_0_ for rotavirus in poor settings such as Bangladesh also results in younger ages at infection, with the result that some infants already have some degree of immunity at the time of vaccination, a factor that might depress measured levels of vaccine protection [28].

If our study findings are generalizable to other low-income settings, where levels of direct protection by RV is lower than reported in affluent countries, it may be unwise for public health leaders to assume that vaccine herd protection will substantially augment the population level protection of RV against ARD. Going forward, in analyses of CRTs to evaluate vaccine herd protection, investigators should be mindful that when the epidemiological assumptions for defining clusters are not fully met and the targeted pathogen is highly transmissible from person to person, the CRT design may not be well suited to measure vaccine herd protection, and even the fried-egg approach may not be adequate to sufficiently compensate for breach of these assumptions.

## Declaration of Competing Interest

The authors declare that they have no known competing financial interests or personal relationships that could have appeared to influence the work reported in this paper.
