# Supplementary material for: Re-evaluation of population-level protection conferred by a rotavirus vaccine using the ‘fried-egg’ approach in a rural setting in Bangladesh
Source: Vaccine. 2021 Sep 24;39(40):5876–82. doi: 10.1016/j.vaccine.2021.08.048 (PMC8494114; doi:10.1016/j.vaccine.2021.08.048)
Supplement: Supplementary data 1 [file mmc1.docx]

**Supplementary Table 1-1**. Baseline characteristics for analysis of overall protection (P75 clusters)

| **Variables** | **RV Villages (N=5007)** | **Non-RV Villages (N=4463)** |
| --- | --- | --- |
| Mean (SD) age at the time of study initiation/migration-in (years) | 0.1 (0.1) | 0.1 (0.1) |
| Male participants (%) | 2537 (51) | 2276 (51) |
| Mother’s education (8-class and above) (%) | 2386 (48) | 2130 (48) |
| Live in a household with a Pacca (%) | 164 (3) | 112 (3) |
| Live in a household with a Television (%) | 1076 (21) | 949 (21) |
| Live in a household with using Septic tank/Modern toilet (%) | 434 (9) | 338 (8) |
| Live in a household using tube well for drinking water (%) | 3616 (72) | 3182 (71) |
| Median (IQR) distance (km) to the icddr,b Matlab hospital | 6.1 (6.5) | 5.6 (4.9) |

Note: the level of significance was derived after adjusting for the design effect; no statistical significance (p<0.05) was detected between RV and non-RV Villages

**Supplementary Table 1-2.** Baseline characteristics for analysis of total protection (P75 clusters)

| **Variables** | **RV Villages (N=3333)** | **Non-RV Villages (N=3594)** |
| --- | --- | --- |
| Mean (SD) age at the time of vaccination date (years)^*^ | 0.2 (0.0) | 0.2 (0.0) |
| Male participants (%) | 1665 (50) | 1842 (51) |
| Mother’s education (8-class and above) (%) | 1535 (46) | 1710 (48) |
| Live in a household with a Pacca (%) | 89 (3) | 88 (2) |
| Live in a household with a Television (%) | 670 (20) | 751 (21) |
| Live in a household with using Septic tank/Modern toilet (%) | 255 (8) | 270 (8) |
| Live in a household using tube well for drinking water (%) | 2418 (73) | 2573 (72) |
| Median (IQR) distance (km) to the icddr,b Matlab hospital | 6.5 (6.0) | 5.6 (4.9) |

Note: the level of significance was derived after adjusted for the design effect; *statistical significance (p<.05) between RV and non-RV Villages

**Supplementary Table 1-3.** Baseline characteristics for analysis of indirect protection (P75 clusters)

| **Variables** | **RV Villages (N=3331)** | **Non-RV Villages (N=3043)** |
| --- | --- | --- |
| Mean (SD) age at the time of study initiation/migration-in (years) | 1.2 (0.5) | 1.2 (0.5) |
| Male participants (%) | 1657 (50) | 1570 (52) |
| Mother’s education (8-class and above) (%) | 1464 (44) | 1311 (43) |
| Live in a household with a Pacca (%) | 137 (4) | 78 (3) |
| Live in a household with a Television (%) | 745 (22) | 694 (23) |
| Live in a household with using Septic tank/Modern toilet (%) | 317 (10) | 233 (8) |
| Live in a household using tube well for drinking water (%) | 2565 (77) | 2341 (77) |
| Median (IQR) distance (km) to the icddr,b Matlab hospital | 6.1 (6.5) | 5.7 (5.0) |

Note: the level of significance was derived after adjusted for the design effect; no statistical significance (p<0.05) was detected between RV and non-RV Villages

**Supplementary Table 2-1**. Baseline characteristics for analysis of overall protection (P50 clusters)

| **Variables** | **RV Villages (N=3407)** | **Non-RV Villages (N=3044)** |
| --- | --- | --- |
| Mean (SD) age at the time of study initiation/migration-in (years) | 0.1 (0.1) | 0.1 (0.1) |
| Male participants (%) | 1710 (50) | 1553 (51) |
| Mother’s education (8-class and above) (%) | 1668 (49) | 1435 (47) |
| Live in a household with a Pacca (%) | 126 (4) | 73 (2) |
| Live in a household with a Television (%) | 781 (23) | 652 (21) |
| Live in a household with using Septic tank/Modern toilet (%) | 317 (9) | 218 (7) |
| Live in a household using tube well for drinking water (%) | 2508 (74) | 2188 (72) |
| Median (IQR) distance (km) to the icddr,b Matlab hospital | 6.1 (6.5) | 5.6 (4.9) |

Note: the level of significance was derived after adjusting for the design effect; *statistical significance (p<.05) between RV and non-RV Villages

**Supplementary Table 2-2.** Baseline characteristics for analysis of total protection (P50 clusters)

| **Variables** | **RV Villages (N=2252)** | **Non-RV Villages (N=2463)** |
| --- | --- | --- |
| Mean (SD) age at the time of vaccination date (years)^*^ | 0.2 (0.0) | 0.2 (0.0) |
| Male participants (%) | 1105 (49) | 1250 (51) |
| Mother’s education (8-class and above) (%) | 1077 (48) | 1153 (47) |
| Live in a household with a Pacca (%) | 71 (3) | 56 (2) |
| Live in a household with a Television (%) | 479 (21) | 519 (21) |
| Live in a household with using Septic tank/Modern toilet (%) | 189 (8) | 175 (7) |
| Live in a household using tube well for drinking water (%) | 1688 (75) | 1780 (72) |
| Median (IQR) distance (km) to the icddr,b Matlab hospital | 6.5 (6.1) | 5.6 (4.9) |

Note: the level of significance was derived after adjusted for the design effect; *statistical significance (p<.05) between RV and non-RV Villages

**Supplementary Table 2-3.** Baseline characteristics for analysis of indirect protection (P50 clusters)

| **Variables** | **RV Villages (N=2249)** | **Non-RV Villages (N=2067)** |
| --- | --- | --- |
| Mean (SD) age at the time of study initiation/migration-in (years) | 1.2 (0.5) | 1.2 (0.5) |
| Male participants (%) | 1122 (50) | 1059 (51) |
| Mother’s education (8-class and above) (%) | 1010 (45) | 874 (42) |
| Live in a household with a Pacca (%) | 92 (4) | 55 (3) |
| Live in a household with a Television (%) | 523 (23) | 483 (23) |
| Live in a household with using Septic tank/Modern toilet (%) | 211 (9) | 163 (8) |
| Live in a household using tube well for drinking water (%) | 1757 (78) | 1598 (77) |
| Median (IQR) distance (km) to the icddr,b Matlab hospital | 6.1 (6.5) | 5.8 (5.0) |

Note: the level of significance was derived after adjusted for the design effect; no statistical significance (p<0.05) was detected between RV and non-RV Villages

**Supplementary Table 3-1**. Baseline characteristics for analysis of overall protection (P25 clusters)

| **Variables** | **RV Villages (N=1778)** | **Non-RV Villages (N=1613)** |
| --- | --- | --- |
| Mean (SD) age at the time of study initiation/migration-in (years) | 0.1 (0.1) | 0.1 (0.1) |
| Male participants (%) | 909 (51) | 836 (52) |
| Mother’s education (8-class and above) (%) | 880 (49) | 752 (47) |
| Live in a household with a Pacca (%) | 56 (3) | 31 (2) |
| Live in a household with a Television (%) | 417 (23) | 349 (22) |
| Live in a household with using Septic tank/Modern toilet (%) | 152 (9) | 123 (8) |
| Live in a household using tube well for drinking water (%) | 1296 (73) | 1185 (73) |
| Median (IQR) distance (km) to the icddr,b Matlab hospital | 6.1 (6.3) | 5.8 (4.9) |

Note: the level of significance was derived after adjusting for the design effect; no statistical significance (p<0.05) was detected between RV and non-RV Villages

**Supplementary Table 3-2.** Baseline characteristics for analysis of total protection (P25 clusters)

| **Variables** | **RV Villages (N=1162)** | **Non-RV Villages (N=1314)** |
| --- | --- | --- |
| Mean (SD) age at the time of vaccination date (years) | 0.2 (0.0) | 0.2 (0.0) |
| Male participants (%) | 583 (50) | 672 (51) |
| Mother’s education (8-class and above) (%) | 562 (48) | 618 (47) |
| Live in a household with a Pacca (%) | 32 (3) | 25 (2) |
| Live in a household with a Television (%) | 254 (22) | 286 (22) |
| Live in a household with using Septic tank/Modern toilet (%) | 95 (8) | 100 (8) |
| Live in a household using tubewell for drinking water (%) | 880 (76) | 970 (74) |
| Median (IQR) distance (km) to the icddr,b Matlab hospital | 6.5 (5.9) | 5.7 (4.9) |

Note: the level of significance was derived after adjusted for the design effect; no statistical significance (p<0.05) was detected between RV and non-RV Villages

**Supplementary Table 3-3.** Baseline characteristics for analysis of indirect protection (P25 clusters)

| **Variables** | **RV Villages (N=1168)** | **Non-RV Villages (N=1078)** |
| --- | --- | --- |
| Mean (SD) age at the time of study initiation/migration-in (years) | 1.2 (0.5) | 1.2 (0.5) |
| Male participants (%) | 585 (50) | 550 (51) |
| Mother’s education (8-class and above) (%) | 534 (46) | 443 (41) |
| Live in a household with a Pacca (%) | 48 (4) | 17 (2) |
| Live in a household with a Television (%) | 269 (23) | 242 (22) |
| Live in a household with using Septic tank/Modern toilet (%) | 114 (10) | 88 (8) |
| Live in a household using tubewell for drinking water (%) | 910 (78) | 835 (77) |
| Median (IQR) distance (km) to the icddr,b Matlab hospital | 6.1 (6.4) | 5.9 (5.1) |

Note: the level of significance was derived after adjusted for the design effect; no statistical significance (p<0.05) was detected between RV and non-RV Villages
